# Effectiveness of Platelet-Rich Fibrin as an Adjunctive Material to Bone Graft in Maxillary Sinus Augmentation: A Meta-Analysis of Randomized Controlled Trails

**DOI:** 10.1155/2019/7267062

**Published:** 2019-03-17

**Authors:** Ruimin Liu, Mingdong Yan, Sulin Chen, Wenxiu Huang, Dong Wu, Jiang Chen

**Affiliations:** ^1^School of Stomatology, Fujian Medical University, Fuzhou, Fujian 350000, China; ^2^Fujian Biological Materials Engineering and Technology Center of Stomatology, Fuzhou, Fujian 350000, China; ^3^Stomatological Key Lab of Fujian College and University, Fujian Medical University, Fuzhou, Fujian 350000, China; ^4^Department of Oral Implantology, Affiliated Stomatological Hospital of Fujian Medical University, Fuzhou, China

## Abstract

**Purpose:**

To date, it remains unknown whether the addition of platelet-rich fibrin (PRF) to bone grafts actually improves the effectiveness of maxillary sinus augmentation. This study aimed to perform a meta-analysis to evaluate the efficacy of PRF in sinus lift.

**Materials and Methods:**

PubMed, Embase, and the Cochrane Library were searched. Randomized controlled studies were identified. The risk of bias was evaluated using the Cochrane Collaboration tool.

**Results:**

Five RCTs were included in our meta-analysis. Clinical, radiographic, and histomorphometric outcomes were considered. No implant failure or graft failure was detected in all included studies within the follow-up period. The percentage of contact length between newly formed bone substitute and bone in the PRF group was lower but lacked statistical significance (3.90%, 95% CI, -2.91% to 10.71%). The percentages of new bone formation (-1.59%, 95% CI, -5.36% to 2.18%) and soft-tissue area (-3.73%, 95% CI, -10.11% to 2.66%) were higher in the PRF group but were not significantly different. The percentage of residual bone graft was not significant in either group (4.57%, 95% CI, 0% to 9.14%).

**Conclusions:**

Within the limitations of this review, it was concluded that there were no statistical differences in survival rate, new bone formation, contact between newly formed bone and bone substitute, percentage of residual bone graft (BSV/TV), and soft-tissue area between the non-PRF and PRF groups. Current evidence supporting the necessity of adding PRF to bone graft in sinus augmentation is limited.

## 1. Introduction

Successful implant restoration is clearly highly dependent on a sufficient bone volume and density. A lack of bone in the posterior maxilla, mainly resulting from the combination of alveolar bone resorption after tooth loss, pneumatization of the maxillary sinus, and periodontal disease, leads to increased difficulty during dental implant treatment. Currently, this problem has been overcome by augmenting the alveolar height via bone grafting followed by maxillary sinus floor lift.

Various materials, such as freeze-dried bone allograft, *β*-calcium phosphate tribasic (*β*-TCP), and bovine bone mineral (DBBM), have been proposed as bone substitutes that are applied to the sinus augmentation procedure [1]. Osteoconductive properties of these bone substitutes have been shown in clinical studies, and satisfactory clinical outcomes [2] can be acquired. However, such bone substitutes lack osteogenic and osteoinductive properties with distinct osteogenic capacities and bone formation rates. Moreover, some disadvantages, mainly related to a prolonged healing time, limited availability, and impact on immune responses, can appear when using these materials.

To overcome these shortcomings, new materials with osteoinductive properties, such as platelet-rich fibrin (PRF) and rhBMP-2, were recently introduced as additional or replacement materials in bone augmentation procedures. The use of biologic mediators with osteoinductive properties has been considered to reduce the time interval and accelerate the formation of new bone [3]. The strengths of PRF in reducing tissue inflammation, promoting the vascularization of bone tissue, accelerating new bone formation, and improving scaffold mechanics have been reported [4].

In recent years, researchers [5–7] have paid greater attention to the clinical results of PRF application in sinus augmentation procedures, but no general consensus has been reached. Some studies have reported positive effects of PRF application in bone augmentation procedures. A recent study [8] detecting the capability of PRF for bone regeneration found that positive effects on bone regeneration could be acquired only when PRF is associated with DBBM. Another preclinical study [9] also indicated that the DBBM-PRF combination resulted in increased angiogenesis and osteogenesis in comparison to DBBM alone. However, other studies have shown limited effects of the efficacy of PRF in bone formation. Zhang et al. [10] found similar effects with DBBM (Bio-Oss) as a sole graft material or with the addition of PRF to Bio-Oss in the sinus floor lift. Knapen et al. [11] indicated that there is no benefit from L-PRF in regard to improving the kinetics, quality, or quantity of bone in guided bone regeneration. They suggested that additional studies considering critical size defect models are needed to confirm their findings.

Varying results have been acquired from the above-mentioned studies, which may puzzle clinicians with contrasting evidence. As no previous studies have conducted a meta-analysis, it is necessary to evaluate the researches on this topic. The aims of our study were to assess the clinical effects of PRF as an adjunctive material to bone graft in sinus augmentation.

## 2. Materials and Methods

Our study was conducted based on the guidelines of the Cochrane Collaboration [12].

### 2.1. Literature Search

The initial electronic searches were performed in Embase, PubMed, and the Cochrane Library (all from inception to March 2018). The search terms (MeSH OR Key Words) “platelet growth factors”, “platelet-rich plasma” (PRP), “platelet concentrate”, “autologous platelet concentrate”, “platelet-rich fibrin”(PRF), “plasma rich growth factors”, “maxillary sinus floor lift”, “maxillary sinus augmentation”, “maxillary sinus floor elevation”, and “dental implants” were used in combination with other strategies to identify potential eligible studies. The publication language was limited to English. To be as inclusive as possible, the search was not restricted by design, region, or publication status. Titles and abstracts of each study were screened to determine articles that should be further evaluated. Full texts of eligible studies were retrieved for further evaluation. In addition, the reference lists of relevant articles were also screened to find other potentially eligible studies. This procedure was conducted by two reviewers (L.-R.M. and Y.-M.D.) independently. Disagreement in relation to enrollment of the retrieved studies was solved by discussion or by consulting another reviewer (C.-S.L.).

### 2.2. Inclusion and Exclusion Criteria

Researches that met the following criteria were eligible for inclusion:

(1) randomized controlled clinical trials assessing the histological and clinical results to reveal the additional effects of PRF in sinus floor augmentation;

(2) studies enrolling human adult subjects.

The exclusion criteria were as follows:

(1) studies involving patients with systemic contraindication or acute maxillary sinusitis or affected by uncontrolled periodontal diseases;

(2) no outcomes of interest;

(3) retrospective, prospective cohort studies, case reports, conference proceedings, and case series; or

(4) duplicate studies.

### 2.3. Data Extraction and Outcome Analyses

Data were collected by two independent review authors (L.-R.M. and Y.-M.D.). Any disagreement concerning exclusion or inclusion of a retrieved study was solved by discussion or by consulting another author (C.-S.L.). If any information was absent, the study authors were contacted for more details. For studies containing the same samples but different periods of follow-up, only the data with the longest follow-up period were extracted. Clinical and radiographic observations included the implant survival rate, complications, and postoperative radiography. Histological results consisted of new bone formation, newly formed bone and bone substitute, percentage of residual bone graft (BSV/TV), and soft-tissue area.

### 2.4. Quality Assessment

Risk of bias of each included study was evaluated by two reviewers (L.-R.M. and Y.-M.D.). Divergence was resolved via discussion and consensus. Cochrane Collaboration's tool was used to assess risk of bias, which consisted of the sequence generation, selection bias, allocation concealment, blinding of participants and personal information, blinding of outcome data, incomplete outcome data, selective reporting, and other biases. Plausible risk of bias for each study was low, moderate, or high. The studies meeting all the criteria were denoted as having a low risk of bias, partly meeting one or more criteria as having a moderate risk of bias, and not meeting one or more criteria as having a high risk of bias (Cochrane Handbook for Systematic Reviews of Interventions, Version 5.1.0, http://handbook-5-1.cochrane.org).

### 2.5. Statistical Analyses

All the meta-analyses were performed using Review Manager 5.2 (Cochrane Collaboration, Oxford, UK). Pooled results for new bone formation, newly formed bone percentage (bone volume per tissue volume, BV/TV), percentage of residual bone graft (BSV/TV), and soft-tissue area and corresponding 95% CIs were applied to assess the clinical and histological effects of PRF in the sinus floor elevation technique. The I^2^ statistic provides a quantitative assessment of the inconsistency among studies, and it is used to determine heterogeneity across studies. The ones with an I^2^ value of 25–50% are judged to have low heterogeneity, those with an I^2^ value of 50–75% have moderate heterogeneity, and those with an I^2^ value >75% have a high degree of heterogeneity [13]. An I^2^ value >50% is considered to represent significant heterogeneity [14]. A pooled analysis was conducted with a random-effect or fixed-effect model based on the heterogeneity determined by the I^2^ test. Furthermore, the risk of publication bias was assessed with a funnel plot.

## 3. Results

**Figure 1:**
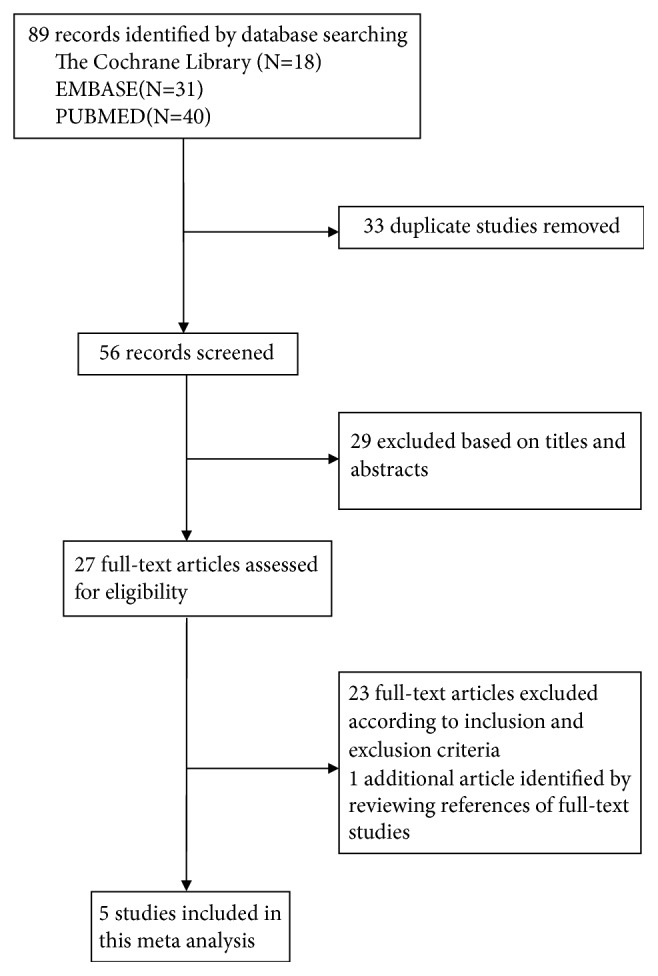
Flowchart of the study identification and selection.

The flowchart for literature selection is shown in Figure 1. Using the outlined search strategy, a total of 89 potentially relevant studies were identified. Thirty-three duplicate studies were eliminated, and another 29 records were excluded based on the title and abstract screening. The remaining 27 studies were further evaluated by reading whole passages. Twenty-three of them were also excluded according to the inclusion and exclusion criteria, while 1 additional article was identified by reviewing the references of the full-text studies. Finally, 5 studies [10, 15–18] were included in this meta-analysis, with 150 sinuses (81 sinuses in the PRF group) enrolled.

### 3.1. Characteristics of the Included Studies

All five included RCTs contained a total of 133 patients, with 81 sinuses in the PRF group and 69 in the control group. All of them were published between 2012 and 2018. The studies were conducted in Italy, China, and Turkey. The main characteristics of the 5 included studies are presented in Table 1. The outcome data for the included articles are presented in Table 2.

### 3.2. Quality Assessment of the Included Studies

**Figure 2:**
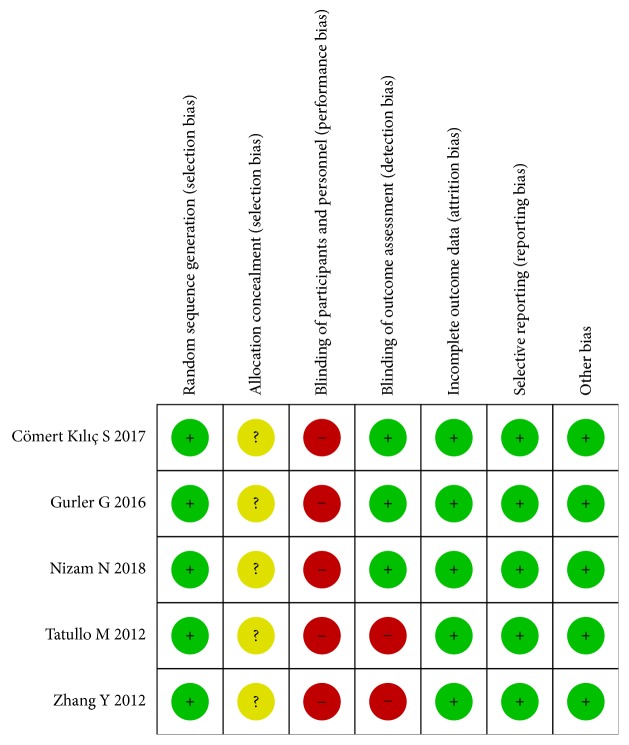
Quality assessment of the included studies. Risk-of-bias summary for the randomized studies (“+” means low risk of bias, “?” means that the risk of bias is unclear, and “−” means a high risk of bias).

We assessed the risk of bias of the included RCTs via a risk-of-bias assessment tool, and the methodological quality assessment is displayed in Figure 2. For the 5 RCTs, blinding of the participants and personnel and allocation concealment were of high risk in all trials. The blinding of outcome assessments was high risk in 2 trials. None of the studies could be identified as having a “low risk bias”. The risk of bias for all 5 studies was “high risk bias”.

### 3.3. Analysis of Outcomes

#### 3.3.1. Clinical and Radiographic Observation

Survival rates of implants were available in only 2 of the included studies [15, 16] during the follow-up period, and the implant survival rates were 100% for both the PRF and non-PRF groups. Two articles [15, 17] reported complications, postoperative bleeding, and Schneiderian membrane perforation, which developed in both augmented sinuses during the surgical and healing period; there was no significant difference between groups among the other studies. One study [18] reported less swelling and pain in the PRF group. Three studies [10, 15, 17] evaluated the postoperative radiography. Evidence for a sufficient amount and density of the bone substitute and bone could be observed in both groups.

#### 3.3.2. Histological Results

Four of 5 included studies reported histological results [10, 15–17]. New bone formation was discovered in both groups. One study [10] reported inflammatory reaction, while no significant signs of an inflammatory reaction could be observed. The other study [16] compared the healing time between groups and indicated that the use of PRF reduced the healing period, supporting optimal bone regeneration. Good primary stability of implants could be achieved at 106 days. PRF application produced, in the “early” protocol, notable neoangiogenesis, thus providing good trophic support for the newly formed bone tissue and leading to more vital bone in comparison to the non-PRF side. One report [17] reported that dense fibrous tissue formation was detected in the platelet concentrate groups, while partly fibrous and cartilaginous tissue formation were detected in the control group.

#### 3.3.3. Histomorphometry

**Figure 3:**
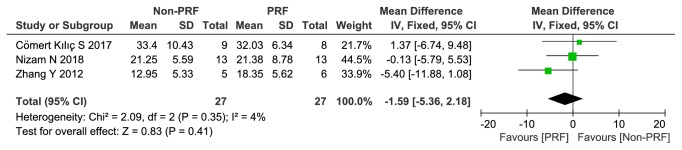
The percentage of new bone formation (%).

*(1) The Percentage of New Bone Formation. *The newly formed bone was characterized as woven bone but not as the mature skeletal tissue of the alveolar crest, which consists of lamellar bone [15]. The percentage of new bone formation in the PRF group was approximately 1.59% higher than in the control group, but this difference was not statistically significant (-1.59, 95% CI -5.36 to 2.18; p=0.41). The I^2^ statistic was 4%, indicating low significant heterogeneity across the studies (Figure 3).

**Figure 4:**
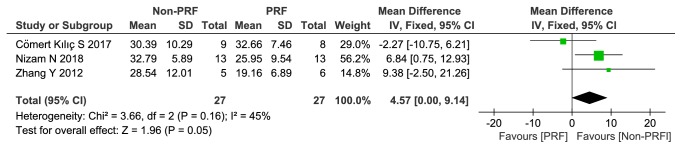
Percentage of residual bone graft (BSV/TV) (%).

*(2) Percentage of Residual Bone Graft (BSV/TV). *There was no significant difference concerning the percentage of residual bone graft (BSV/TV) between the groups, with 4.57% less residual bone graft observed in the PRF compared with the control group (*95*%* CI 0.00 to 9.14; p=0.05). *As I^2^ was less than 50%, a fixed-effects model was selected (x^2^ = 3.66, P =0.16, I^2^ = 45%) (Figure 4). The details of each study can also be found in Figure 4.

**Figure 5:**
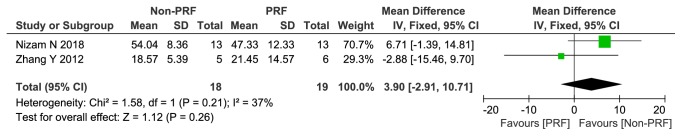
The percentage of contact between newly formed bone substitute and bone (%).

*(3) The Percentage of Contact between Newly Formed Bone Substitute and Bone. *The data for the contact between the newly formed bone substitute and bone were extracted from two studies. Although the percentage for the PRF group was 3.9% less than the control group, no statistically significant difference was detected (3.90, 95% CI -2.91 to 10.71; p=0.26). The fixed-effects model was selected due to acceptable heterogeneity (I^2^= 37%, p= 0.21) (Figure 5).

**Figure 6:**
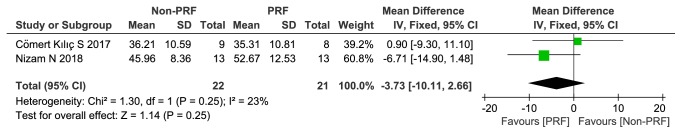
The percentage of the soft-tissue area (%).

*(4) Soft-Tissue Area Percentage. *The soft-tissue area percentage was higher in the PRF group than the non-PRF group (3.73%, 95% CI -10.11 to 2.66; p=0.25), but no significant difference and low heterogeneity were detected (x^2^ = 1.30, P =0.25, I^2^ = 23%) (Figure 6).

## 4. Discussion

Sinus floor elevation has frequently been considered a standard procedure for achieving sufficient bone height and volume in the severely atrophic posterior maxilla [19].Various bone substitutes have been proposed to sustain the lifted space. Growth factors, derived from centrifugation of autologous whole blood, have been used in sinus augmentation either as a sole filling material or in combination with bone substitute materials [20].

The PRF protocol was first described by Dohan et al. [21] and applied to maxillary sinus augmentation in 2006. At present, researchers pay more attention to the application of growth factors due to the multiple advantages offered by this approach. First, platelet-rich fibrin (PRF) has the capability to gradually release autologous growth factors during the first 7 days [22] and shows a gradual decrease during the following 28 days; PRF presents a stronger and more durable effect on the differentiation and proliferation of osteoblasts than PRP in vitro [23, 24]. Second, PRF can be easily reshaped to form a membrane that serves as a matrix to accelerate wound healing, improve new bone formation, and reduce healing period of graft materials. Third, PRF is easy to prepare and manipulate, and it is inexpensive. [25] Patients receiving dental implants after this sinus floor augmentation surgery with PRF technology showed a 100% survival rate with a mean follow-up of 33 months. [26] In addition, Dohan et al. [21] showed that PRF also plays a crucial role in suppressing inflammatory reactions, thus acting as a node of immune regulation; these effects were attributed to the release of anti-inflammatory cytokines. Trisi et al. [27] determined that platelet-rich fibrin glue, in combination with autogenous bone and Biogran, could improve new bone formation and that a greater amount of bone formation could be observed after only five to six months. Because of the above-mentioned advantages, PRF was considered for use as graft particles in combination with bone substitute materials in maxillary sinus floor augmentation. Combinations of bone substitute material and PRF in sinus augmentation have been reported in clinical and animal studies; however, the effects have remained uncertain and have puzzled clinicians. The present meta-analysis was designed to assess the current literature on the clinical and histological results of PRF application for sinus augmentation.

Following wide and restricted literature search and selection, 5 RCTs were included in our study. The results of our meta-analysis seem to suggest that PRF does not provide additional benefits compared with non-PRF groups in regard to bone formation. The survival rate of the implants and complications were the two primary outcomes. For the limited number of included studies, only two articles reported implant survival conditions, and the follow-up periods were relatively short. No uncontrolled complications developed in either augmented sinus during the healing period. Our results are in accordance with previous findings. Fabbro et al. [28] conducted a systematic review and showed that positive effects on soft-tissue healing and less postoperative discomfort were commonly reported but not quantified. Based on a histological evaluation, Tatullo et al. [16] concluded that PRF application reduced the healing time, cutting the time interval to 120 days compared with the 150 days described in the literature. As only one study reported related results, a quantitative analysis was not conducted.

A recent animal study [29] compared the resorption rates among DBBM, PRF, and biphasic calcium phosphate. The results showed a much higher resorption rate for PRF but a reduced total bone volume at 6 months in comparison to the other groups. Conversely, our study showed that the percentage of new bone formation was 1.59% higher in the PRF group than the control group, although this difference was not statistically significant.

In our study, four histological results were included for quantitative analysis. The results of the quantitative analysis revealed insignificant differences regarding the percentages of contact length between newly formed bone substitute and bone, newly formed bone, residual graft particles, and soft-tissue areas between the two groups (P> 0.05). These results may indicate that the use of PRF as an adjunctive material to bone graft does not actually improve the amount of regenerated bone and is not superior in comparison to the control groups.

Similar conclusions have been acquired in former systematic reviews examining the efficacy of autologous growth factor application in sinus floor lift. Fabbro et al. [28] concluded that a clear advantage of platelet concentrate application in sinus floor lift could not be evidenced. Rickert et al. [30] found that the addition of growth factors (platelet-rich plasma) to autogenous bone did not promote bone formation.

Currently, platelet concentrate and stem cells (SCs) are both used in tissue engineering. Platelets make up the frontline healing response to injury because they release growth factors for tissue repair. SCs can differentiate into specific types of cells and tissues. SCs also produce some growth factors and cytokines that accelerate the healing process of sites of tissue damage [31].

The proliferation and expansion of mesenchymal stem cells (MSCs) produce a large number of potential osteoblasts, while the properties of MSCs vary from different subpopulations. Stem cell surface markers may help to select the most potent population of MSCs for regenerative medical applications [32]. However, if a bone defect is only filled with MSCs, it may require a large amount. For platelet concentrate, a long-term and effective role in the process of osteogenesis would be difficult because of rapid absorption. A previous animal study [33] was designed to combine dM-SCs (dog mesenchymal stem cells) with growth factors for the repair of bone defects. This combination resulted in more rapid and effective bone regeneration, suggesting a positive influence of PRP on MSCs. Therefore, future research might further explore this phenomenon.

In our study, clinical trial registries and unpublished gray literature search were not utilized because it is difficult to derive data from such documents. Moreover, due to limited articles, no funnel plot was used to detect publication bias. Such limitations may potentially affect the results of our review.

In general, heterogeneity could not be avoided in the meta-analyses. In contrast, some other limitations should also be considered when explaining the present results. First, only a limited number of RCTs were enrolled in this meta-analysis, which may have reduced the power in detecting significant differences. Second, methodological shortcomings could theoretically lead to low power and potential bias. Most of the included studies described methods of blinding and allocation concealment that were not adequate. None of the included studies could be judged to have a low risk of bias. Our quality assessment showed that risk of bias in all enrolled studies was high. The influence of the high risk of bias on study outcomes is difficult to quantify, but such methodological shortcomings should be considered when interpreting the results of this systematic review.

Inter-study heterogeneity might be influenced by the following factors: differences in the PRF preparation production, different grafting materials, and eligibility criteria, among others. The FDA-CE approved system is Intra-Spin L-PRF (Intra-Lock, Boca Raton, USA). However, some products that are “PRF-like” but differ from the original L-PRF can be found in articles, which may have contributed to the high heterogeneity. As reported by Ehrenfest et al. [34], the centrifuge characteristics and centrifugation protocols significantly and dramatically impact the cells, growth factors, and fibrin architecture of L-PRF.

Moreover, we cannot overlook the influence of other related factors on maxillary sinus lift surgery with bone grafting. For example, tobacco smoking is considered to be a risk factor for periodontal and general health [35]. Smoking might reduce the implant success rate [36] and increase the risk of maxillary sinus mucosa damage. In addition, nicotine may induce contraction of the peripheral blood vessels, resulting in a decreased osteogenic capacity [37].

Third, language restriction may play a role in the publication bias, as only studies published in English were included in our study.

In conclusion, although the addition of PRF to bone substitutes may help to reduce the healing time, its use as an adjunctive material does not seem to actually improve the effectiveness of sinus augmentation; the results of our meta-analysis indicate an absence of differences in the survival rate, new bone formation, contact between newly formed bone and bone substitute, percentage of residual bone graft (BSV/TV), and soft-tissue area between non-PRF and PRF groups. PRF preparation is time-consuming, and blood drawing may contribute to patient discomfort. Therefore, the use of PRF as an adjunctive material to bone grafting in sinus augmentation is not currently recommended for routine use due to the limited evidence. Additionally, PRF preparation techniques were different in the included studies, which may contribute a large bias. A strong conclusion concerning the present results remains difficult. Future well-designed RCTs with long-term follow-ups including the same version of PRF are required to substantiate our findings due to the present study limitations.

## Figures and Tables

**Table 1a:** (a) Characteristics of the included studies

First Author (Year of Publication)	Study Design	Study Location	Population (Mean age)	Female/ Male	Intervention
Nizam N (2018)	RCT, SM	School of Dentistry, Ege University, İzmir, Turkey	Thirteen patients (mean age ± SD: 49.92 ± 10.37)	4/9	Bio-Oss and L-PRF mixture (test) Bio-Oss alone (control)

Zhang Y (2012)	RCT, P	Department of Implant Dentistry, Peking University, School and Hospital of Stomatology, Beijing, China	The test group: six sinuses from six patients (mean age, 43.5 years; range, 30-49) the control group: five sinuses of five patients (mean age, 46.2 years; range, 37-53).	2/8	Bio-Oss and L-PRF mixture (test) Bio-Oss alone (control)

Comert K S, 2017	RCT, P	Department of Oral and Maxillofacial Surgery, Center for Oral and Dental Health, Erzurum, Turkey	26 patients: Ages ranged from 22-51 years The control group: 31.51±8.52 years The P-PRP group: 34.01±9.59 years The PRF group: 35.48±9.53 years	9 /17	B-TCP (control); P-PRP mixed B-TCP (The P-PRP group); PRF mixed B-TCP (PRF group).

Tatullo M, 2012	RCT, P	Dept. of Basic Medical Science, University of Bari, Italy	60 patients, 43 and 62 years	48/12	Deproteinized bovine bone (Bio-Oss) PRF+deproteinized bovine bone (Bio-Oss)

Gurler G, 2016	RCT, P	Department of Oral and Maxillofacial Surgery, İstanbul Medipol University School of Dentistry, Atatürk Bulvarı	24 patients: age ranged from 23-66 years (mean age 47.8) The study group: mean age of 46.3 years The control group: mean age of 49.3 years	10/14	Allogenous bone + L-PRF (test) Allogenous bone (control)

RCT: randomized controlled trial.

SM: split-mouth.

P: prospective.

**Table 1b:** (b) Characteristics of the included studies

First Author (Year of Publication)	Outcomes	Follow-up	PRF Preparation technique	RBH (mm)
Nizam N, 2018	Primary outcomes: newly formed bone, residual bone graft, and newly formed bone-to-bone contact Secondary outcomes: clinical and radiographic data (6 months after augmentation; implant survival rate 12 months after implant loading)	12 months	A table centrifuge (Nüve Laboratory Equipment, NF200, Ankara, Turkey) or 12 min at 400 × g	Test: 2.45 ± 0.79 Control: 2.53± 0.61

Zhang Y, 2012	New bone formation: percentage of residual bone substitute contact length between newly formed bone and bone substitute	6 months	PRF by Choukroun's Procedure (300 g for 10 min)	< 5

Comert K S, 2017	Primary outcomes: new bone formation, mean percentages of residual graft particle area, percentages of soft tissue, and no postoperative maxillary sinus infection Secondary outcomes: mean densities of bone cells (osteoblasts, osteoclasts, osteocytes, and osteoprogenitors), capillary vessels, and inflammatory cells	Control group: 6.14±0.57 months; P-PRP group: 6.08±0.67 months; PRF group: 6.29±0.53 months	PRF by Choukroun's Procedure (3000 rpm for 10 min)	< 7

Tatullo M, 2012	New bone; complications; ISQ values (Implant Stability Quotient); bone density; clinical success rate	150 days	Choukroun's procedure	< 5

Gurler G, 2016	Healing Index (HI) scores Inflammatory and infectious reactions Patient comfort	14 days	Centrifuge (IntraSpin, USA) at 2700 rpm for 12 min	NA

RBH: residual bone height.

**Table 2a:** (a) Outcome data of randomized controlled trials of the included studies.

Study	Year	Implant number		Sinus		Residual bone height (mm)		New bone formation		Residual graft particles		Soft tissue area	
		N—PRF	PRF	N—PRF	PRF	N—PRF	PRF	N—PRF	PRF	N—PRF	PRF	N—PRF	PRF
Nizam N	2018	28	30	13	13	2.53±0.61	2.45±0.79	21.25±5.59%	21.38± 8.78%	32.79± 5.89%	25.95 ±9.54%	45.96±8.36%	52.67±12.53%

Zhang Y	2012	Delayed implant placement		5	6	NA	NA	12.95±5.33%	18.35 ± 5.62%	28.54 ±12.01%	19.16±6.89%	NA	NA

Comert K S	2017	Delayed implant placement		9	8	NA	NA	33.40±10.43%	32.03±6.34%	30.39 ±10.29%	32.66 ±7.46%	36.21 ±10.59%	35.31 ±10.81%

Tatullo M	2012	Delayed implant placement		30	42	NA	NA	NA	NA	NA	NA	NA	NA

Gurler G	2016	Delayed implant placement		12	12	NA	NA	NA	NA	NA	NA	NA	NA

N-PRF: non-platelet-rich fibrin group; PRF: platelet-rich fibrin group; NA: not available. The percentage of newly formed bone (BV/TV); the percentage of residual bone graft (BSV/TV); the percentage of length of the profile of the bone graft in contact with new bone (bone-to-bone graft contact).

**Table 2b:** (b) Outcome data of randomized controlled trials of the included studies.

Study	Year	Contact between newly formed bone and bone substitute		Augmented bone Height (mm)		Survival rate		Complications (N—PRF/PRF)
		N—PRF	PRF	N—PRF	PRF	N—PRF	PRF	
Nizam N	2018	54.04 ± 8.36%	47.33± 12.33%	13.53±1.20	13.60± 1.09	100%	100%	Bleeding (1/1) Sinus perforation (0/0)

Zhang Y	2012	18.57±5.39%	21.45± 14.57%	NA	NA	NA	NA	Inflammation (0/0)

Comert K S	2017	NA	NA	NA	NA	NA	NA	Schneiderian membrane perforation (2/2) No sinus infection

Tatullo M	2012	NA	NA	NA	NA	100%	100%	None

Gurler G	2016	NA	NA	NA	NA	NA	NA	Less swelling and pain in the PRF group

N-PRF: non-platelet-rich fibrin group; PRF: platelet-rich fibrin group; NA: not available. The percentage of newly formed bone (BV/TV); the percentage of residual bone graft (BSV/TV); the percentage of length of the profile of the bone graft in contact with new bone (bone-to-bone graft contact).
